# The Content of Phenolic Compounds in *Stevia rebaudiana* (Bertoni) Plants Derived from Melatonin and NaCl Treated Seeds

**DOI:** 10.3390/plants12040780

**Published:** 2023-02-09

**Authors:** Magdalena Simlat, Agata Ptak, Tomasz Wójtowicz, Agnieszka Szewczyk

**Affiliations:** 1 Department of Plant Breeding, Physiology and Seed Science, University of Agriculture in Krakow, Łobzowska 24, 31-140 Krakow, Poland; 2 Department of Pharmaceutical Botany, Faculty of Pharmacy, Jagiellonian University Medical College, Medyczna 9, 30-688 Krakow, Poland

**Keywords:** flavonoids, melatonin, NaCl, *Stevia rebaudiana*, phenolic acids

## Abstract

Stevia is a plant with many beneficial properties. It contains not only steviol glycosides, which are used as non-caloric natural sweeteners, but also a number of metabolites with antioxidant properties. This study examined the content of both phenolic acids and flavonoids in stevia leaves as an effect of treating seeds with melatonin and conducting germination in NaCl conditions. The results of our research indicated higher amounts of phenolic acids compared to flavonoids in stevia leaves. Among these acids, isochlorogenic, rosmarinic, and chlorogenic acids were accumulated in the largest amounts, regardless of the germination conditions. For 5 and 100 µM of melatonin treatments, the content of both phenolic acids and flavonoids increased. However, in salinity conditions (50 mM NaCl), 500 µM of melatonin had the most favorable effect on the synthesis of phenolic acids. The phenolic acids in that case reached a level three-times higher than that in the samples with the same melatonin concentration but without NaCl. We also found that the content of phenolic compounds varied depending on the age of the leaves. To the best of our knowledge, this is the first study to describe the effect of melatonin and NaCl on the synthesis on phenolic acids and flavonoids in stevia.

## 1. Introduction

*Stevia rebaudiana* (Bertoni) is a perennial herb belonging to the Asteraceae family. The natural areas of occurrence of stevia are Paraguay and Brazil. Currently, the population of stevia in natural sites is significantly limited, mainly due to the excessive grazing of animals and plant exploitation [1]. Interest in stevia results mainly from the content of steviol glycosides (SGs), which are sweet, zero-calorie metabolites that can successfully replace traditional sugar in human diets. These compounds are derivatives of tetracyclic diterpenes, and the main part of their molecules is steviol linked by glycosidic bonds with the sugar residue [2]. About 30 SGs have been identified in stevia; these differ in the number of sugar elements, with the main sweet-tasting compounds being stevioside and rebaudioside A [3,4,5,6,7,8]. Steviol glycosides are found mainly in leaves, and their synthesis begins in plastids along the methylerythritol phosphate pathway (MEP). In leaves, the estimated concentrations of stevioside is 6.5–9.1% and rebaudioside A is 2.3–3.8% [9,10]. Smaller amounts are also found in the stem and flowers. The SG content varies depending on the stage of plant development. Angelini et al. [8] showed that the greatest amounts of SGs accumulate in the leaves during the flowering period. Steviol glycosides are not only natural sweeteners, they also have proven health-promoting properties; since they lower the glycemic index, they are especially recommended for people with diabetes. The sweetening properties of stevia have been known for a long time, but it was not until the 20th century that SGs were approved for use in the human diet by the EU Commission [11].

In recent years, there has been an increase in the number of studies and published works on stevia. This interest has resulted not only from the plant’s sweetening properties but also from its antibacterial [12,13,14,15], antiviral [16], anticancer [17,18], and antioxidant [19,20] abilities. As in other plants, these antioxidant effects are partly due to the presence of phenolic acids and flavonoids [20,21]. To date, more than 30 phenolic compounds have been reported in stevia plants [22,23,24,25,26,27,28,29,30]. The major phenolic compounds from stevia leaves are chlorogenic acid, isochlorogenic acid, and other hydroxycinnamic acids [31].

Melatonin (MEL; N-acetyl-5-methoxytryptamine) is a multiple-function molecule that was first identified in animals and later in plants [32]. Melatonin in plants regulates versatile processes involved in growth and development, including seed germination, root architecture, flowering time, leaf senescence, fruit ripening, and biomass production. There is already evidence that treating seeds in MEL solutions prior to sowing increases not only germination but also plant growth [33,34,35,36,37,38,39,40,41,42,43,44,45,46]. The effects of melatonin under various abiotic and biotic stress conditions have also been observed [41,47,48,49,50]. Recently published studies have also shown that MEL affects secondary metabolism in plants [46,51,52].

Although various approaches for applying MEL have been tested, there is no research related to phenolic acid and flavonoid contents in *S. rebaudiana* as a result of seed treatment. Therefore, this study is the first to describe the content of phenolic compounds in stevia plants derived from seeds treated with various concentrations of MEL and additionally germinated on NaCl.

## 2. Results and Discussion

### 2.1. Phenolic Acids and Flavonoids Identified in Stevia Leaves

**Figure 1:**
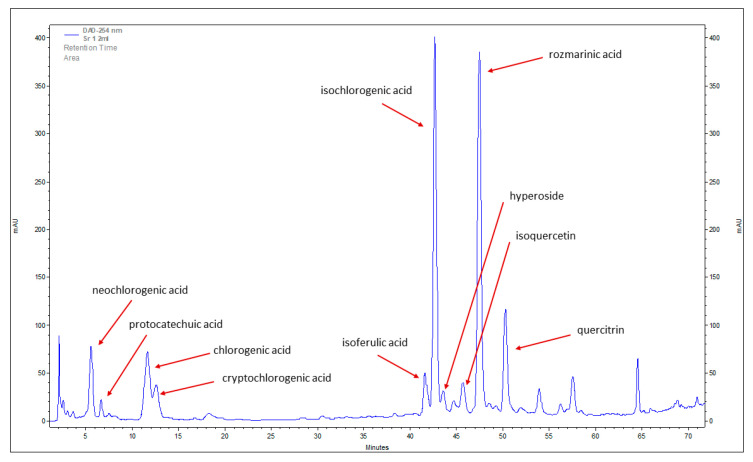
Reverse-phase high performance liquid chromatography (RP-HPLC) chromatogram of phenolic acids and flavonoids identified in methanolic extract of stevia leaves (the leaves were from plants derived from control seeds—incubated in water before germination experiment).

Using reverse-phase high performance liquid chromatography (RP-HPLC) of methanolic extract of stevia leaves, regardless of how the seeds were treated, we were able to identify seven phenolic acids—chlorogenic, neochlorogenic, protocatechuic, cryptochlorogenic, isoferulic, isochlorogenic, and rosmarinic—and three flavonoids—hyperoside, isoquercetin, and quercitrin (Figure 1; Table 1). However, we did not identify any catechins. Previously, Karaköse et al. [22] reported 24 chlorogenic acids in a chloroform-methanolic extract of *S. rebaudiana* leaves. These chlorogenic acids were mainly presented as hydroxycinnamic acid derivatives of quinic and shikimic acid. Rajbhandari and Roberts [53] described six flavonoid glycosides in Stevia nepetifolia: apigenin-4′-*O*-glucoside, luteolin-7-*O*-glucoside, kaempferol-3-*O*-rhamnoside, quercitrin, quercitin-3-*O*-glucoside, and quercetin-3-*O*-arabinoside. Marchyshyn et al. [54] indicated the presence of five flavonoids in *S. rebaudiana* leaves: rutin, hyperoside, luteolin, quercetin-3-D-glycoside, and kaempferol. Carrera-Lanestosa [55] also found luteolin, quercetin, and apigenin in *S. rebaudiana*. The differences in the qualitative composition of the identified compounds can be explained by the source of the analyzed plant material and the growth condition. There is also evidence that the method of extraction also affects the pool of identified compounds [31,56,57].

### 2.2. The Effect of Melatonin on Phenolic Acid and Flavonoid Content

**Figure 2:**
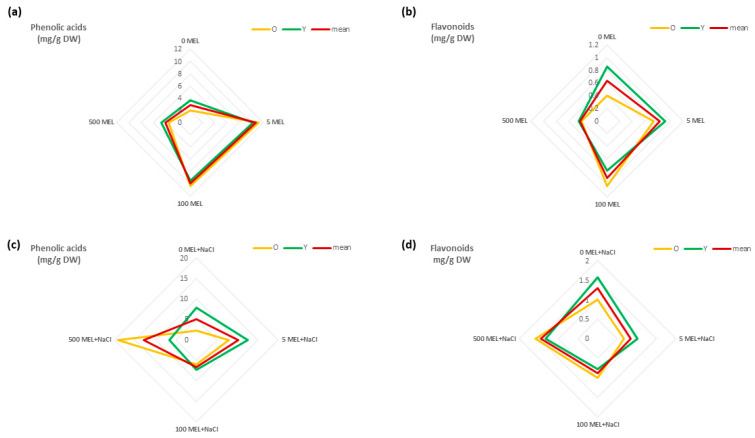
The content (mg/g of dry weight [DW]) of the sum of phenolic acids (**a**,**c**) and flavonoids (**b**,**d**) in methanolic extracts of stevia leaves under melatonin (**a**,**b**) and melatonin/NaCl (**c**,**d**) treatments. 0 MEL, 5 MEL, 100 MEL, 500 MEL—different concentrations of melatonin: 0 μM, 5 μM, 100 μM, and 500 μM, respectively.

In previous research, we indicated the effect of MEL treatment of seeds on the development and growth of stevia plants as well as on the synthesis of SGs [46]. For this study, we investigated its effects on the contents of phenolic acids and flavonoids. As previously indicated, MEL could enhance the level of flavonoids and different forms of isoflavone during soybean germination [58]. Xu et al. [59] demonstrated that melatonin treatment increased the contents of total phenols (for 18 of 22 phenolic compounds), flavonoids, anthocyanins, and proanthocyanidins in berries. Ptak et al. [52] found that MEL enhanced the galanthamine biosynthesis in the in vitro culture of *Leucojum aestivum*, with the most promising effect shown for the concentration of 5 µM of MEL. In the experiment presented here, treatment of seeds before germination with 5 μM and 100 μM of MEL also increased the contents of total phenolic acids (5.5-fold and 5.2-fold, respectively) and total flavonoids (1.9-fold and 2.6-fold, respectively) in stevia leaves compared to those in the control. A higher concentration of MEL (500 μM) had no such beneficial effects (Figure 2a,b). Regardless of the concentration of MEL, the total content of phenolic acids was higher than that of flavonoids, which is consistent with the results of other studies [23,60].

In this study, the content of phenolic compounds were analyzed separately in older and younger stevia leaves. Nevertheless, we did not observe any effect of the leaf maturity stage on the total phenolic acid content. Despite this, Liu et al. [61] previously showed that younger leaves of tea plants contained twice as much phenolic acid as older ones. However, we did find that the stage of leaf development had an effect on the content of flavonoids in relation to the concentration of MEL. In older leaves, we observed the highest combined amounts of the three flavonoids (hyperoside, isoquercetin, and quercitrin) for 100 μM of MEL. In younger and control leaves, these compounds accumulated in the highest amounts for plants derived from seeds treated with 5 µM of melatonin. The highest concentration of melatonin (500 μM) used for seed treatment had no effect on the synthesis of the determined flavonoids in stevia leaves (Figure 2a,b).

Despite no differences in the qualitative composition of phenolic acids and flavonoids, on the basis of the peak area of each compound, we found that melatonin concentrations and the maturity stage of the leaves significantly affected the content of individual phenolic compounds (Table 2).

Padda and Picha [62] also described the different levels of certain phenolic acids in sweet potato leaves at different stages of maturity. However, no studies have shown the effect of melatonin on the content of individual phenolic compounds and flavonoids. In our research, the highest phenolic acid concentrations were found for isochlorogenic, rosmarinic, and chlorogenic acids, and the highest flavonoid concentration was for quercitrin. The average content of each of these compounds was 3.18, 2.38, and 0.62 of dry weight (DW) for isochlorogenic, rosmarinic, and chlorogenic acids, respectively, and 0.58 mg/g for quercitrin (Table 3).

Regarding isochlorogenic and chlorogenic acids, their concentrations were significantly higher in younger leaves compared to older ones in all tested samples with 5 μM of MEL. The contents of chlorogenic acid was 1.9-fold and isochlorogenic acid was 3.3-fold higher compared to those in the control. With an increase in MEL concentration, the content of both acids decreased slightly for both older and younger leaves. Our research confirms the literature indicating that the main phenolic acids in stevia leaves are isochlorogenic and chlorogenic acid [22,31]. However, this is the first study to show the influence of MEL on the biosynthesis of these acids. Regarding rosmarinic acid, when MEL was used for the seed treatment before germination, it significantly increased the content in older leaves compared to that in younger leaves at all tested concentrations; however, this effect was best seen with lower melatonin concentrations (Table 3). Among the identified flavonoids, quercitrin was accumulated in the greatest amount. In that case, the highest concentration (about 2.7-times higher than that in the control) was detected in older leaves under 100 μM of MEL (0.87 mg/g of DW). However, 500 μM of MEL significantly decreased the quercitrin concentration, specifically in younger leaves (Table 3).

The effect of MEL on plants seems to be long-term [63]. Therefore, a specific concentration of MEL used for seed treatment before germination may be a good stimulator of the biosynthesis of phenolic compounds in *S. rebaudiana* leaves. For the extraction of specific compounds, attention should be paid to the use of leaves in the appropriate maturity stage.

### 2.3. The Effect of Melatonin on Phenolic Acid and Flavonoid Content under NaCl

Salinity is one of the most significant abiotic factors that negatively influences plant efficiency and the production of many crops. Around 19.5% of irrigated agricultural land is considered to be saline [64]. Stevia plantlet growth decreases as salinity increases [65,66,67]. Our previous study also showed that stevia germination and growth is impacted by salt stress [46]. Although a low level of salinity (50 mM) had a positive impact on the morphological parameters, 150 mM of sodium chloride (NaCl) exerted an unfavorable effect on seedling development, resulting in growth inhibition and even death [46]. Our previous study also indicated that 50 mM of NaCl increased the stevioside content but decreased rebaudioside A [46]. This finding was in agreement with other research showing that low levels of NaCl increased SG, chlorophyll a, carotenoids, and total sugars [68,69]. However, no previous studies have shown the effect of NaCl on the content of individual phenolic compounds in stevia. In this study, we found that NaCl at a concentration of 50 mM increased the contents of both phenolic acids and total flavonoids (Figure 2c,d). Similarly, Zhu et al. [70] found that in *Taraxacum officinale*, the contents of chlorogenic and isochlorogenic acids were higher under salt stress conditions (≤1 g kg^−1^) compared to those in the control, with the opposite effect observed at higher concentrations of NaCl. An interesting solution limiting the negative impact of NaCl is the use of MEL. Melatonin is a molecule with a wide spectrum of activity, and in many species, it has been proven to have a beneficial effect on growth under stress conditions, including salt stress. In *Malus hupehensis* L., exogenous melatonin modulated the flavonoid content in response [71]. Bistgani et al. [72] found that foliar application of MEL improved the total phenolic compounds in *Thymus daenensis* L. leaves under salinity stress. In this study, we observed that the treatment of seeds with 5 or 500 μM of MEL before germination on 50 mM of NaCl affected the total phenolic acid concentration in stevia leaves. However, 5 μM of MEL had the best effect on younger leaves, while 500 μM of MEL had the best effect on older leaves (Figure 2c). Additionally, the highest sum of flavonoids was observed for 500 μM of MEL. We observed that 500 μM of MEL increased the total flavonoid content in older leaves, while 5 µM and no melatonin (0 MEL) increased the content in younger leaves (Figure 2d). In a previous study [73,74], melatonin affected the concentrations of the flavonoids and total phenolic acids. In our research, MEL also had a significant effect on the content of individual phenolic compounds under NaCl conditions (Table 2). The highest accumulations were recorded for isochlorogenic, rosmarinic, and chlorogenic acids (average contents of 3.46, 2.78, and 0.81 mg/g of DW, respectively) as well as quercitrin (0.72 mg/g of DW). The highest contents of rosmarinic and isochlorogenic acids were recorded in older leaves obtained from seeds exposed to 500 µM of MEL (9.28 and 7.74 mg/g of the DW, respectively), which were about 13-times and 10-times higher, respectively, than their amounts in the older leaves of control plants (Table 3). The results described above indicate that in the presence of NaCl, melatonin regulates the content of individual phenolic compounds in a slightly different way compared to conditions without NaCl.

### 2.4. The Effect of Melatonin and NaCl on the Accumulation of Phenolic Compounds

**Figure 3:**
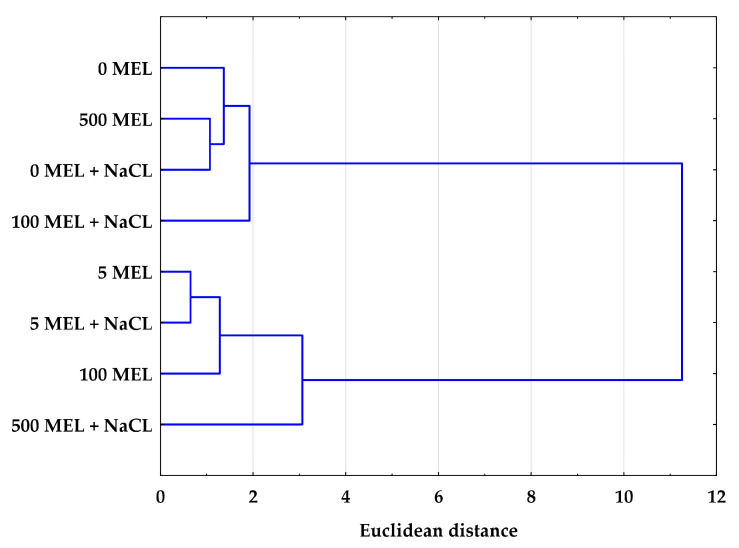
The dendrogram based on Ward’s hierarchical clustering based on the average content of the identified phenolic compounds in stevia leaves. Stevia leaves were obtained from plants derived from melatonin treated seeds and germinated in control or in 50 mM NaCl conditions (+NaCl) (0 MEL, 5 MEL, 100 MEL, 500 MEL—different concentrations of melatonin used for seed treatment: 0 μM, 5 μM, 100 μM, and 500 μM, respectively).

To examine the linkage between the effects of melatonin and NaCl in terms of the studied biochemical compounds, Ward’s [75] hierarchical clustering was applied. Based on the average content of the identified phenolic compounds, two clusters formed on the dendrogram (Figure 3). The similarities shown in both clusters indicate the important roles of both melatonin and NaCl in inducing a specific response in secondary metabolism. At a lower melatonin concentration (5 μM) and in the absence of melatonin (0 MEL), the presence of NaCl had no significant effect on the content of phenolic compounds in stevia plants. However, the presence of NaCl in the germination medium had an effect on seeds treated with melatonin at higher concentrations (100 and 500 μM); samples that germinated in the presence and absence of NaCl in the medium were located in different clusters (Figure 3). This may indicate that in addition to the adverse effects of NaCl observed in many plant species, higher concentrations of melatonin may also induce a stress response in stevia plants [76].

**Figure 4:**
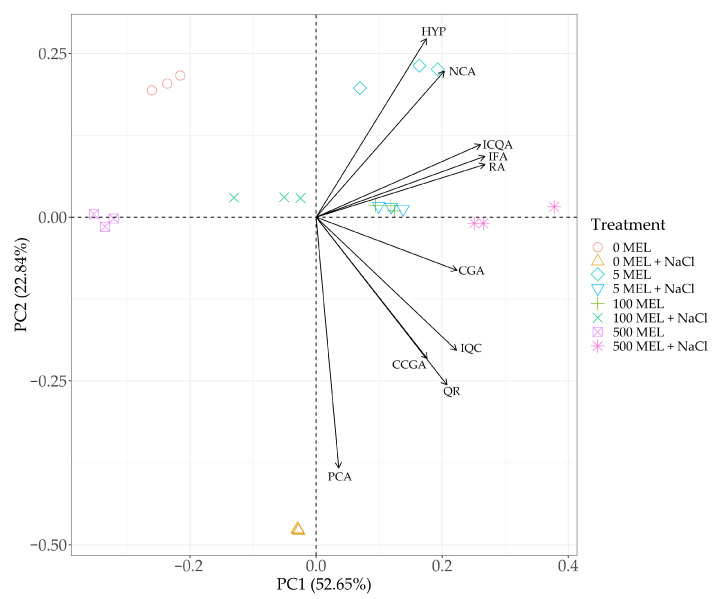
Principal component analysis (PCA) biplot showing relationships between the melatonin and NaCl treatment in the context of phenolic compound contents in stevia leaves: neochlorogenic acid (NCA), protocatechuic acid (PCA), chlorogenic acid (CGA), cryptochlorogenic acid (CCGA), isoferulic acid (IFA), isochlorogenic acid (ICQA), hyperoside (HYP), isoquercetin (IQC), rosmarinic acid (RA), and quercitrin (QR). Stevia leaves were obtained from plants derived from melatonin treated seeds and germinated in control or in 50 mM NaCl conditions (+NaCl) (0 MEL, 5 MEL, 100 MEL, 500 MEL—different concentrations of melatonin used for seed treatment: 0 μM, 5 μM, 100 μM, and 500 μM, respectively).

Principal component analysis (PCA) showed that the first component (PC1) explained nearly 53% of the variation, while the second component (PC2) explained about 23% of the variation. PC1 was significantly affected by almost all variables except the protocatechuic acid content. In contrast, PC2 was strongly negatively influenced by the content of protocatechuic acid, cryptochlorogenic acid, quercitrin, and isoquercetin. However, the contents of neochlorogenic acid and hyperoside had a significant positive effect on PC2 (Figure 4). The position of the loading vectors for the individual variables made it possible to indicate several relationships. A significant positive correlation was found between the contents of neochlorogenic acid and hyperoside. Additionally, a significant relationship was found between the contents of isochlorogenic, isoferulic, and rosmarinic acids. A significant relationship was also seen between the contents of cryptochlorogenic acid and quercitrin and the content of isoquercetin. The biplot chart indicates the relationship between the studied treatments and the content of the analyzed phenolics. From the distribution of the data, it can be concluded that the control, characterized by the absence of melatonin treatment and NaCl, was not related to the variation in any of the analyzed acids. In contrast, the plants treated only with NaCl were characterized by a particular increase in protocatechuic acid content not observed in other treatments. Melatonin at a dose of 5 μM was specifically associated with an increase in the content of neochlorogenic acid and a higher content of hyperoside. A similar distribution of phenolic compound contents was found in the plants obtained from seeds treated with MEL at a dose of 100 μM and at a dose of 500 μM in combination with NaCl, especially for chlorogenic, isochlorogenic, isoferulic, and rosmarinic acids (Figure 4).

## 3. Materials and Methods

### 3.1. Plant Material

The stevia plants were obtained from seeds incubated in an aqueous solution of MEL prior to germination.

The seeds were surface-sterilized according to Simlat et al. [77] before treatment. Three concentrations of MEL were used: 5 μM (5 MEL), 100 μM (100 MEL), and 500 μM (500 MEL). For the control, the seeds were incubated in water (0 MEL). After incubation, half of the seeds were germinated in in vitro conditions on agar gel and the other half on agar gel supplemented with 50 mM of NaCl. After three weeks of germination experiment, seedlings were transferred to the Murashige and Skoog (MS) [78] medium for one month and then well-developed plantlets were transplanted into pots (15 × 15 cm) containing soil, peat, and sand in a 3:1:1 ratio. The plants used for analysis were grown for six months in controlled conditions (25 °C, fluorescent light with intensity expressed as Photosynthetic Photon Flux Density of 320 μmol m^−^^2^s^−^^1^ for 16 h/day, and 70% ± 5% relative humidity; Adaptis-A1000AR, Conviron, Winnipeg, MB, Canada).

### 3.2. RP-HPLC Analysis

The leaves of six-month-old plants were used for the RP-HPLC analysis. Phenolic acids, flavonoids, and catechins were quantified in methanol extracts obtained by sonification (30 °C, 1 h) of 250 mg of dry biomass in 2 mL of methanol. RP-HPLC analyses were conducted according to the method described by Ellnain-Wojtaszek and Zgórka [79] with modifications using a Merck–Hitachi liquid chromatograph (LaChrom Elite, Hitachi, Tokyo, Japan) equipped with a DAD detector L-2455 and Purospher **^®^** RP-18e (250 × 4 mm/5 mm) column (Merck, Darmstadt, Germany). Analyses were carried out at 25°C with a mobile phase consisting of A—methanol and B—methanol: 0.5% acetic acid; 1:4 (*v*/*v*). The gradients were as follows: 100% B for 0–20 min, 100–80% B for 20–35 min, 80–60% B for 35–55 min, 60–0% B for 55–70 min, 0% B for 70–75 min, 0–100% B for 75–80 min, and 100% B for 80–90 min at a flow rate of 1 mL min-1 and λ = 254 nm for phenolic acids and catechins and λ = 370 nm for flavonoids. Identification was done by comparison of the retention times of the peaks with authentic reference compounds and co-chromatography with standards. Quantification was done by measurement of the peak area with reference to the standard curve derived from five concentrations (0.03125 to 0.5 mg ml^−^^1^). Caffeic, chlorogenic, cinnamic, ellagic, gallic, gentisic, isoferulic, neochlorogenic, *o*-coumaric, protocatechuic, rosmarinic, salicylic, sinapic, syringic acid, apigetrin (apigenin 7-glucoside), hyperoside (quercetin 3-*O*-galactoside), isoquercetin (quercetin 3-*O*-glucoside), isorhamnetin, kaempferol, luteolin, populin (kaempferol 7-*O*-glucoside), quercetin, quercitrin (quercetin 3-*O*-rhamnoside), rhamnetin, rutin, and vitexin standards were purchased from Sigma-Aldrich (St Louis, MO, USA). Vanillic, *p*-coumaric, ferulic, and *p*-hydroxybenzoic acid standards were purchased from Fluka (Bucha, Switzerland). Cryptochlorogenic acid, isochlorogenic acid, catechin, epigallocatechin, epicatechin gallate, epicatechin, epigallocatechin gallate, and cynaroside (luteolin 7-*O*-glucoside) standards were purchased from ChromaDex (Irvine, CA, USA).

### 3.3. Statistical Analyses

The data were reported as mean ± SD. To study the effect of MEL and NaCl on the phenolic acid and flavonoid content, factorial ANOVA (TIBCO Statistica, Palo Alto, CA, USA) was applied. To estimate the significance of differences between the means, we performed Duncan’s multiple range test at a significance level of *p* < 0.05. To examine the linkage between the effects of MEL and NaCl in terms of the studied biochemical compounds, Ward’s [75] hierarchical clustering was applied. The dendrogram was generated based on the phenolic acid and flavonoid contents. To determine the relationship between treatment and the phenolic content, the data were analyzed with PCA using R v. 3.6.1 [80].

## 4. Conclusions

Taking into account the present results as well as previously published studies on *S. rebaudiana*, it is reasonable to conclude that stevia is an abundant source of secondary metabolites with health benefits. The presence of bioactive compounds in stevia leaves belonging to phenolic and flavonoid groups tend to justify their medicinal properties and their application in both the food and pharmaceutical industries.

Furthermore, our studies pointed out that MEL used at the stage of seed germination seems to be a good stimulator of the biosynthesis of phenolic compounds in stevia plants, and low dose of NaCl may be an additional stimulating factor. This has a very practical aspect: MEL treated seeds can be used in the establishment of plantations for obtaining high quality plants useful for the food and pharmaceutical industries.

Although significant progress has been made in understanding the role of MEL in plants, the MEL signaling pathway under salt stress remains unclear. Melatonin’s mitigation of salt stress damages can be achieved through multiple mechanisms: (1) MEL reduces excessive production of reactive oxygen species (ROS) by facilitating electron leakage as a result of increasing the efficiency of the mitochondrial electron transport chain; (2) MEL regulates ion homeostasis; (3) MEL modulates the activity of transcription factors; and (4) MEL may also mitigate cell damage and reduce the increase in osmotic pressure caused by salt stress by increasing the accumulation of the phenolic compounds and all phenolic acids.

Comprehensive and well-designed future research on the role of MEL in biosynthesis of phenolic compounds under salt stress in *S. rebaudiana* would be valuable. Our further research will focus on the expression of genes involved in the metabolism of phenylpropanoids and the analysis of the activity of relevant enzymes. We believe that these analyses will by valuable for understanding these mechanisms. Such knowledge could create new opportunities to optimize the biosynthesis of these medicinally important metabolites.

## Figures and Tables

**Table 1:** Phenolic compounds detected in stevia leaves by LC/UV, LC/MS, and LC/ESI/MS.

Compound	Retention Time (t_r_ min)	Formula	Molecular Mass (R_r_)
neochlorogenic acid	5.88	C_16_H_18_O_9_	354.31
protocatechuic acid	6.98	C_7_H_6_O_4_	154.12
chlorogenic acid	11.12	C_16_H_18_O_9_	354.31
cryptochlorogenic acid	12.63	C_16_H_18_O_9_	354.31
isoferulic acid	39.32	C_10_H_10_O_4_	194.18
isochlorogenic acid	42.33	C_16_H_18_O_9_	354.31
hyperoside	43.94	C_21_H_20_O_12_	464.38
isoquercetin	45.43	C_21_H_20_O_12_	464.38
rosmarinic acid	47.16	C_18_H_16_O_8_	360.31
quercitrin	49.9	C_21_H_20_O_11_	448.38

**Table 2:** Variance analysis for the content of phenolic compounds with regard to melatonin concentration and the maturity of stevia leaves. The analyses were carried out separately for plants obtained from seeds treated with melatonin and germinated under control conditions (−NaCl) and for plants obtained from melatonin treated seeds and germinated on a medium with the addition of NaCl (+NaCl).

Compound	Source	df	MS	F–Value	MS	F–Value
			–NaCL		+NaCl	
**neochlorogenic acid**	MEL concentration (M)	3	0.277576	223.510 ***	0.040497	45.423 ***
	Type of leaves (T)	1	0.015395	12.397 **	0.017980	20.167 ***
	M × T	3	0.009742	7.844 **	0.101441	113.781 ***
	Error	16	0.001242		0.000892	
**protocatechuic acid**	M	3	0.001284	162.482 ***	0.006266	1140.207 ***
	T	1	0.000442	55.952 ***	0.000432	78.547 ***
	M × T	3	0.000066	8.398 **	0.000087	15.741 ***
	Error	16	0.000008		0.000005	
**chlorogenic acid**	MEL concentration (M)	3	0.583775	181.817 ***	0.20679	33.541 ***
	Type of leaves (T)	1	0.779998	242.931 ***	4.09826	664.724 ***
	M × T	3	0.049906	15.543 ***	0.54243	87.981 ***
	Error	16	0.003211		0.00617	
**cryptochlorogenic acid**	MEL concentration (M)	3	0.112743	364.286 *	0.040865	77.858 ***
	Type of leaves (T)	1	0.000318	1.029 *^ns^*	0.016176	30.819 ***
	M × T	3	0.000863	2.789 *^ns^*	0.051125	97.408 ***
	Error	16	0.000309		0.000525	
**isoferulic acid**	MEL concentration (M)	3	18.7137	323.605 ***	0.079112	116.3412 ***
	Type of leaves (T)	1	4.9781	86.083 ***	0.087028	127.9835 ***
	M × T	3	0.1069	1.849 ***	0.092553	136.1074 ***
	Error	16	0.0578		0.000680	
**isochlorogenic acid**	MEL concentration (M)	3	0.060263	216.828 ***	15.7731	113.472 ***
	Type of leaves (T)	1	0.033797	121.603 ***	3.4197	24.601 ***
	M × T	3	0.009105	32.762 *^ns^*	19.6256	141.186 ***
	Error	16	0.000278		0.1390	
**rosmarinic acid**	MEL concentration (M)	3	15.7940	292.521 ***	20.4823	104.381 ***
	Type of leaves (T)	1	5.5199	102.234 ***	18.9816	96.732 ***
	M × T	3	1.6759	31.040 ***	21.1283	107.672 ***
	Error	16	0.0540		0.1962	
**hyperoside**	MEL concentration (M)	3	0.002441	77.082 ***	0.001401	31.794 ***
	Type of leaves (T)	1	0.000001	0.025 *^ns^*	0.002616	59.368 ***
	M × T	3	0.000842	26.589 ***	0.002523	57.248 ***
	Error	16	0.000032		0.000044	
**isoquercetin**	MEL concentration (M)	3	0.005569	286.379 ***	0.030799	243.874 ***
	Type of leaves (T)	1	0.008554	439.882 ***	0.020344	161.088 ***
	M × T	3	0.000727	37.401 ***	0.002065	16.355 ***
	Error	16	0.000019		0.000126	
**quercitrin**	MEL concentration (M)	3	0.175491	199.646 ***	0.32960	81.646 ***
	Type of leaves (T)	1	0.028423	32.335 ***	0.03266	8.089 *
	M × T	3	0.113650	129.292 ***	0.19715	48.838 ***
	Error	16	0.000879		0.00404	

*ns*, not significant; * significant at the 0.05 probability level; ** significant at the 0.01 probability level; *** significant at the 0.001 probability level.

**Table 3:** The content of individual phenolic acids and flavonoids (mg/g of DW) in stevia leaves obtained from melatonin and NaCl treated seeds. Results are presented as a mean of three replications (*n* = 3) ± SD. Different letters in each column (separately for −NaCl and +NaCl) indicate a significant difference of *p* < 0.05 according to Duncan’s test; for mean value of each compound, homogeneous groups were designated separately and marked with capital letters. 0 MEL, 5 MEL, 100 MEL, 500 MEL—different concentrations of melatonin used for seed treatment: 0 μM, 5 μM, 100 μM, and 500 μM, respectively; O—older leaves, Y—younger leaves. Monitored compounds: neochlorogenic acid (NCA); protocatechuic acid (PCA); chlorogenic acid (CGA); cryptochlorogenic acid (CCGA); isoferulic acid (IFA); isochlorogenic acid (ICQA); rosmarinic acid (RA); hyperoside (HYP); isoquercetin (IQC); quercitrin (QR).

MEL Concentration	Type of Leaves	Phenolic Acids							Flavonoids		
		NCA	PCA	GCA	CCGA	ICQA	IFA	RA	HYP	IQC	QR
−**NaCl**											
**0 MEL**	**O**	0.209 ^c^ ± 0.05	0.018 ^e^ ± 0.00	0.238 ^f^ ± 0.06	0.128 ^c^ ± 0.03	0.716 ^f^ ± 0.15	0.037 ^de^ ± 0.01	0.664 ^f^ ± 0.17	0.059 ^d^ ± 0.01	0.031 ^f^ ± 0.01	0.313 ^e^ ± 0.04
	**Y**	0.367 ^b^ ± 0.01	0.018 ^e^ ± 0.00	0.640 ^cd^ ± 0.01	0.138 ^c^ ± 0.00	1.694 ^e^ ± 0.04	0.035 ^e^ ± 0.00	0.770 ^ef^ ± 0.01	0.094 ^a^ ± 0.00	0.086 ^c^ ± 0.00	0.685 ^c^ ± 0.03
	**mean**	0.288 ^C^	0.018 ^C^	0.440 ^B^	0.133^C^	1.205 ^D^	0.306 ^B^	0.717 ^B^	0.076 ^A^	0.059 ^B^	0.499 ^BC^
**5 MEL**	**O**	0.609 ^a^ ± 0.08	0.043 ^bc^ ± 0.00	0.628 ^d^ ± 0.09	0.274 ^b^ ± 0.03	4.722 ^b^ ± 0.52	0.307 ^a^ ± 0.04	4.555 ^a^ ± 0.58	0.082 ^b^ ± 0.01	0.065 ^d^ ± 0.01	0.588 ^d^ ± 0.04
	**Y**	0.581 ^a^ ± 0.04	0.033 ^d^ ± 0.00	1.229 ^a^ ± 0.11	0.247 ^b^ ± 0.02	5.542 ^a^ ± 0.34	0.139 ^c^ ± 0.01	2.481 ^c^ ± 0.02	0.067 ^cd^ ± 0.00	0.118 ^b^ ± 0.01	0.738 ^b^ ± 0.03
	**mean**	0.595 ^A^	0.038 ^B^	0.929 ^A^	0.261 ^B^	5.132 ^A^	0.223 ^A^	3.518 ^A^	0.075 ^A^	0.092 ^A^	0.663 ^AB^
**100 MEL**	**O**	0.337 ^b^ ± 0.00	0.055 ^a^ ± 0.00	0.738 ^c^ ± 0.02	0.368 ^a^ ± 0.01	3.799 ^c^ ± 0.01	0.271 ^b^ ± 0.01	4.819 ^a^ ± 0.03	0.071 ^c^ ± 0.00	0.092 ^c^ ± 0.00	0.868 ^c^ ± 0.02
	**Y**	0.397 ^b^ ± 0.01	0.045 ^b^ ± 0.00	0.939 ^b^ ± 0.02	0.396 ^a^ ± 0.01	4.404 ^b^ ± 0.10	0.160 ^c^ ± 0.01	3.188 ^b^ ± 0.10	0.068 ^cd^ ± 0.00	0.129 ^a^ ± 0.00	0.578 ^a^± 0.01
	**mean**	0.367 ^B^	0.050 ^A^	0.838 ^A^	0.382^A^	4.101 ^B^	0.215 ^A^	4.003 ^A^	0.070 ^A^	0.110 ^A^	0.723 ^A^
**500 MEL**	**O**	0.068 ^d^ ± 0.01	0.055 ^a^ ± 0.01	0.160 ^f^ ± 0.02	0.066 ^d^ ± 0.00	1.668 ^e^ ± 0.18	0.066 ^d^ ± 0.01	1.402 ^d^ ± 0.10	0.041 ^e^ ± 0.00	0.040 ^e^ ± 0.01	0.322 ^e^ ± 0.03
	**Y**	0.081^d^ ± 0.00	0.040 ^c^ ± 0.00	0.398 ^e^ ± 0.03	0.083 ^d^ ± 0.00	2.907 ^d^ ± 0.08	0.047 ^de^ ± 0.00	1.165 ^de^ ± 0.06	0.026 ^f^ ± 0.00	0.047 ^e^ ± 0.00	0.365 ^e^± 0.01
	**mean**	0.074 ^D^	0.048 ^A^	0.279 ^B^	0.075 ^D^	2.287 ^C^	0.057 ^B^	1.284 ^B^	0.034 ^B^	0.044 ^B^	0.343 ^C^
**+NaCl (50 mM)**											
**0 MEL**	**O**	0.017 ^e^ ± 0.00	0.091 ^a^ ± 0.00	0.301 ^f^ ± 0.00	0.234 ^c^ ± 0.00	0.780 ^e^ ± 0.00	0.039 ^e^ ± 0.00	0.694 ^e^ ± 0.00	0.037 ^d^ ± 0.00	0.143 ^d^ ± 0.00	0.821 ^c^ ± 0.00
	**Y**	0.377 ^b^ ± 0.00	0.092 ^a^ ± 0.00	1.530 ^b^ ± 0.00	0.548 ^a^ ± 0.00	3.303 ^c^ ± 0.00	0.097 ^d^ ± 0.00	1.982 ^d^ ± 0.00	0.045 ^cd^ ± 0.00	0.215 ^b^ ± 0.00	1.323 ^a^ ± 0.00
	**mean**	0.197 ^B^	0.091 ^A^	0.915 ^A^	0.391 ^A^	2.041 ^C^	0.068 ^C^	1.338 ^B^	0.041 ^B^	0.179 ^B^	1.072 ^A^
**5 MEL**	**O**	0.331 ^b^ ± 0.02	0.040 ^c^ ± 0.00	0.619 ^e^ ± 0.04	0.367 ^b^ ± 0.03	2.932 ^c^ ± 0.21	0.164 ^b^ ± 0.01	3.394 ^b^ ± 0.25	0.052 ^c^ ± 0.00	0.094 ^e^ ± 0.01	0.531 ^d^ ± 0.03
	**Y**	0.446 ^a^ ± 0.02	0.029 ^e^ ± 0.00	1.942 ^a^ ± 0.12	0.365 ^b^ ± 0.02	6.514 ^b^ ± 0.31	0.150 ^bc^ ± 0.01	3.108 ^b^ ± 0.15	0.069 ^b^ ± 0.00	0.178 ^c^ ± 0.01	0.777 ^c^ ± 0.03
	**mean**	0.389 ^A^	0.035 ^C^	1.281 ^A^	0.366 ^AB^	4.723 ^AB^	0.157 ^AB^	3.251 ^AB^	0.061 ^AB^	0.136 ^BC^	0.653 ^B^
**100 MEL**	**O**	0.265 ^c^ ± 0.05	0.019 ^f^ ± 0.00	0.580 ^e^ ± 0.13	0.258 ^c^ ± 0.05	2.034 ^d^ ± 0.37	0.106 ^cd^ ± 0.02	2.801 ^bc^ ± 0.61	0.069 ^b^ ± 0.01	0.094 ^e^ ± 0.01	0.824 ^c^ ± 0.11
	**Y**	0.275 ^c^ ± 0.01	0.012 ^g^ ± 0.00	1.327 ^c^ ± 0.06	0.271 ^c^ ± 0.01	3.456 ^c^ ± 0.18	0.069 ^de^ ± 0.00	1.923 ^d^ ± 0.09	0.035 ^d^ ± 0.00	0.097 ^e^ ± 0.00	0.639 ^d^ ± 0.03
	**mean**	0.270 ^AB^	0.015 ^D^	0.953 ^A^	0.264 ^BC^	2.745 ^BC^	0.088 ^C^	2.362 ^B^	0.052 ^AB^	0.095 ^C^	0.732 ^B^
**500 MEL**	**O**	0.464 ^a^ ± 0.05	0.051 ^b^ ± 0.01	0.872 ^d^ ± 0.10	0.276 ^c^ ± 0.02	7.739 ^a^ ± 0.89	0.565 ^a^ ± 0.07	9.285 ^a^ ± 1.05	0.113 ^a^ ± 0.02	0.226 ^b^ ± 0.03	1.247 ^a^ ± 0.13
	**Y**	0.197 ^d^ ± 0.00	0.034 ^d^ ± 0.00	0.880 ^d^ ± 0.00	0.158 ^d^ ± 0.00	3.231 ^c^ ± 0.00	0.077 ^de^ ± 0.00	2.046 ^cd^ ± 0.00	0.041 ^cd^ ± 0.00	0.300 ^a^ ± 0.00	0.981 ^b^ ± 0.00
	**mean**	0.330 ^AB^	0.043 ^B^	0.876 ^A^	0.217 ^C^	5.485 ^A^	0.321 ^A^	5.665 ^A^	0.077 ^A^	0.263 ^A^	1.114 ^A^

## Data Availability

The data presented in this study are available on request from the corresponding author.
